# The Marine-Derived Kinase Inhibitor Fascaplysin Exerts Anti-Thrombotic Activity

**DOI:** 10.3390/md13116774

**Published:** 2015-11-09

**Authors:** Emmanuel Ampofo, Thomas Später, Isabelle Müller, Hermann Eichler, Michael D. Menger, Matthias W. Laschke

**Affiliations:** 1 Institute for Clinical and Experimental Surgery, Saarland University, 66421 Homburg/Saar, Germany; E-Mails: tomcali@icloud.com (T.S.); michael.menger@uks.eu (M.D.M.); matthias.laschke@uks.eu (M.W.L.); 2 Institute for Hemostasiology and Transfusion Medicine, Saarland University, 66421 Homburg/Saar, Germany; E-Mails: isabelle.müller@uks.eu (I.M.); hermann.eichler@uks.eu (H.E.)

**Keywords:** fascaplysin, thrombosis, platelets, GPIIb/IIIa, P-selectin, leukocytes, CD11b, dorsal skinfold chamber

## Abstract

Background: The marine-derived kinase inhibitor fascaplysin down-regulates the PI3K pathway in cancer cells. Since this pathway also plays an essential role in platelet signaling, we herein investigated the effect of fascaplysin on thrombosis. Methods: Fascaplysin effects on platelet activation, platelet aggregation and platelet-leukocyte aggregates (PLA) formation were analyzed by flow cytometry. Mouse dorsal skinfold chambers were used to determine *in vivo* the effect of fascaplysin on photochemically induced thrombus formation and tail-vein bleeding time. Results: Pre-treatment of platelets with fascaplysin reduced the activation of glycoprotein (GP)IIb/IIIa after protease-activated receptor-1-activating peptide (PAR-1-AP), adenosine diphosphate (ADP) and phorbol-12-myristate-13-acetate (PMA) stimulation, but did not markedly affect the expression of P-selectin. This was associated with a decreased platelet aggregation. Fascaplysin also decreased PLA formation after PMA but not PAR-1-AP and ADP stimulation. This may be explained by an increased expression of CD11b on leukocytes in PAR-1-AP- and ADP-treated whole blood. In the dorsal skinfold chamber model of photochemically induced thrombus formation, fascaplysin-treated mice revealed a significantly extended complete vessel occlusion time when compared to controls. Furthermore, fascaplysin increased the tail-vein bleeding time. Conclusion: Fascaplysin exerts anti-thrombotic activity, which represents a novel mode of action in the pleiotropic activity spectrum of this compound.

## 1. Introduction

Fascaplysin is a marine-derived kinase inhibitor that was originally isolated from the Fijian marine sponge *Fascaplysinopsis* [1]. Over the last decade, several studies suggested the application of this compound in cancer therapy, because it exerts multiple anti-tumor effects. For instance, fascaplysin is cytotoxic via intercalation into the DNA [2] and induction of apoptotic cell death by inhibition of cyclin-dependent-kinase-4/cyclin D1 (CDK4/D1) [1]. Moreover, fascaplysin inhibits the proliferation of human umbilical vein endothelial cells (HUVEC), suppresses VEGF expression and reduces capillary plexus formation in the chorioallantoic membrane model, indicating an anti-angiogenic activity [3,4]. Finally, Kumar *et al.* [5] recently reported that fascaplysin down-regulates PI3K/AKT/mTOR signaling in cancer cells. Noteworthy, this central pathway is not only essential for cell survival and cell growth, but also plays a crucial role in thrombus formation [6,7,8,9].

Thrombus formation is a complex process, which involves the interaction of soluble plasma components, platelets and leukocytes. The molecular mechanisms of platelet activation are strictly regulated by several phospho-regulated pathways, including PI3K/AKT signaling [10,11,12,13]. All PI3K isoforms are expressed in platelets and are responsible for the generation of phosphatidylinositol 3,4,5-trisphosphate (PIP3) via phosphorylation of phosphatidylinositides in the plasma membrane [12]. This, in turn, stimulates protein kinase B (PKB/Akt) by a conformational switch, resulting in platelet activation [8,12,14].

Consequently, several surface proteins are up-regulated on platelets, including glycoprotein (GP)IIb/IIIa and P-selectin [15,16,17]. The activated form of GPIIb/IIIa recognizes RGD-containing glycoproteins, such as fibrinogen or vWF, which mediates platelet aggregation and effective hemostatic plug formation [18,19,20]. On the other hand, P-selectin binds to its counter-receptor P-selectin glycoprotein ligand (PSGL)-1 on leukocytes, resulting in the additional incorporation of platelet-leukocyte aggregates (PLA) in the newly developing thrombus [16,21,22].

Based on the important role of PI3K/AKT in platelet signaling and the observed inhibitory effect of fascaplysin on this pathway in cancer cells, we herein hypothesized that fascaplysin may exert potent anti-thrombotic activity. To test this hypothesis, we first examined *in vitro* the effect of the compound on platelet activation, platelet aggregation and PLA formation. Moreover, dorsal skinfold chambers in BALB/c mice were used to analyze *in vivo* the effect of fascaplysin on photochemically induced thrombus formation and tail-vein bleeding time.

## 2. Results

### 2.1. Effect of Fascaplysin on Platelet Viability and PI3K Signaling

**Figure 1:**
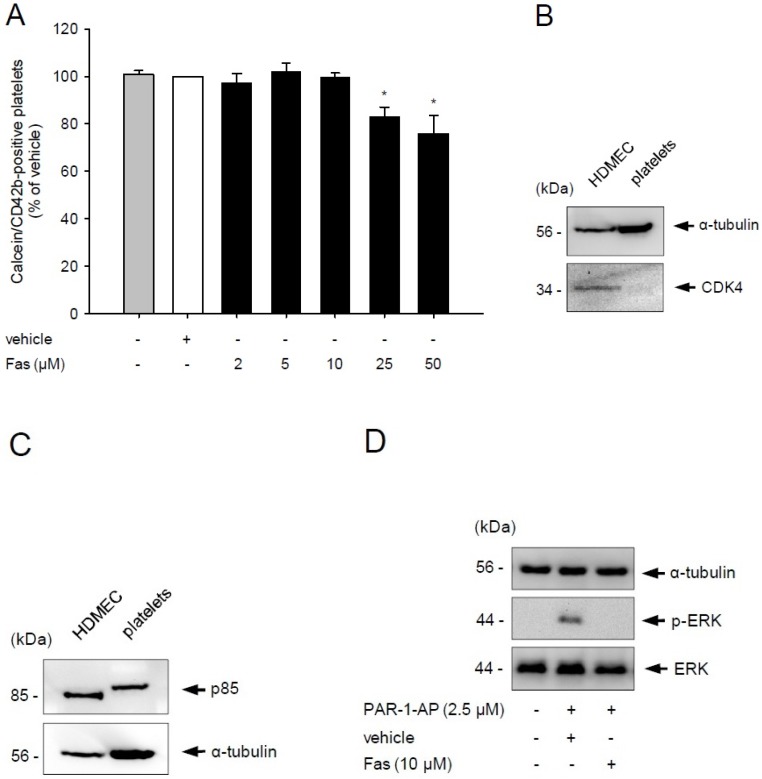
Effect of fascaplysin on platelet viability and PI3K signaling. (**A**) Washed platelets (WP) were incubated with different concentrations of fascaplysin (black bars, *n* = 4) or vehicle dimethyl sulfoxide (DMSO), white bars, *n* = 4) for 0.5 h. Untreated platelets served as negative control (grey bars, *n* = 4). Platelet viability was assessed by flow cytometry. Data are given in % of vehicle. Mean ± SEM. * *p* < 0.05 *vs.* vehicle; (**B**,**C**) Western blot analysis of CDK4, p85 and α-tubulin expression in untreated human dermal microvascular endothelial cells (HDMEC) and WP. The shown Western blots are representative of experiments conducted in triplicate; (**D**) Western blot analysis of ERK, p-ERK and α-tubulin expression in WP, which were incubated with 10 μM fascaplysin for 0.5 h followed by stimulation with protease-activated receptor-1-activating peptide (PAR-1-AP). The shown Western blots are representative of experiments conducted in triplicate.

In a first set of experiments, we analyzed the effect of fascaplysin on platelet viability by flow cytometry. We found that fascaplysin doses up to 10 μM did not exert any toxic effects, whereas higher concentrations of 25 and 50 μM significantly reduced the viability of platelets (Figure 1A). Based on these results, we decided to use only fascaplysin doses up to 10 μM for all following *in vitro* assays to investigate the effects of the kinase inhibitor in a non-toxic dose range.

Fascaplysin suppresses PI3K signaling in cancer cells [5]. Moreover, fascaplysin has been shown to exert a strong inhibitory activity on CDK4 (IC_50_ = 0.35 μM) [1,4]. Besides, it also inhibits CDK6, for which encoding mRNA levels could be previously detected in platelets [23]. However, the inhibitory activity of fascaplysin on CDK6 is much lower (IC_50_ = 3.4 μM) [24]. Therefore, we decided to investigate the level of CDK4 and PI3K in washed platelets by Western blot analysis. Human dermal microvascular endothelial cells (HDMEC) served as controls. As expected, HDMEC expressed CDK4, because these cells have a nucleus and undergo a regular cyclin-controlled cell cycle. In contrast, platelets are anucleated, non-proliferating cells and, thus, they did not express CDK4 (Figure 1B). However, they exhibited a strong expression of the PI3K subunit p85 (Figure 1C). Therefore, we further analyzed whether fascaplysin reduces PI3K activity in platelets by assessing ERK phosphorylation, which is involved in downstream signaling of PI3K [25]. Our Western blot analyses revealed that washed platelets stimulated with protease-activated receptor-1-activating peptide (PAR-1-AP) exhibit an increased phosphorylation of ERK, indicating an activation of the PI3K pathway (Figure 1D). In contrast, treatment with 10 μM fascaplysin markedly reduced this phosphorylation (Figure 1D).

### 2.2. Effect of Fascaplysin on Platelet Activation

**Figure 2:**
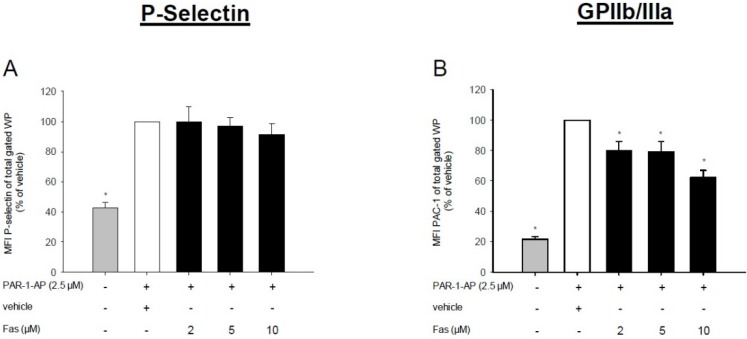

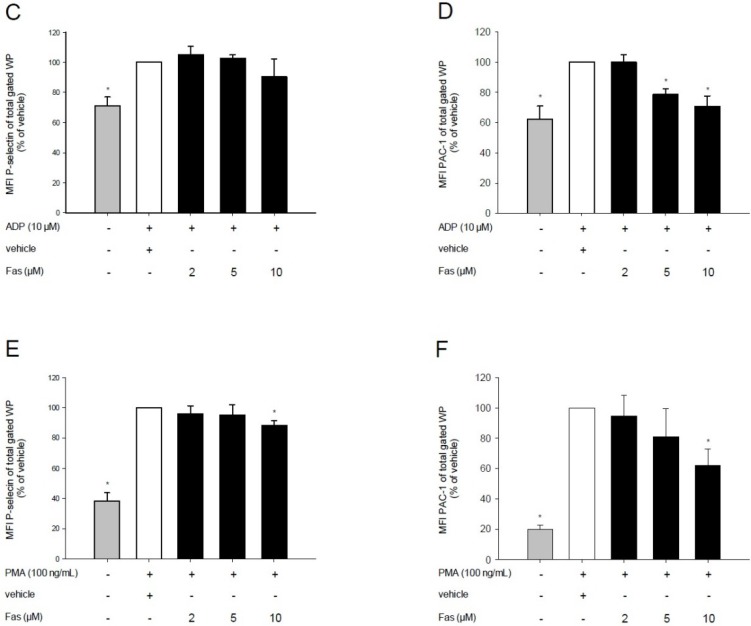
Effect of fascaplysin on agonist-induced P-selectin expression and GPIIb/IIIa activation. (**A**–**F**) WP were incubated with different concentrations of fascaplysin (black bars, *n* = 6) or vehicle (DMSO, white bars, *n* = 6) for 0.5 h following stimulation with PAR-1-AP (**A**,**B**), adenosine diphosphate (ADP) (**B**,**D**), or phorbol-12-myristate-13-acetate (PMA) (**E**,**F**). Data are given in % of vehicle. Unstimulated platelets served as negative control (grey bars, *n* = 6). Surface levels of PAC-1 (recognizing the activated form of glycoprotein (GP)IIb/IIIa) and P-selectin were assessed by flow cytometry. Mean ± SEM. * *p* < 0.05 *vs.* vehicle.

In a next step, we investigated the effect of fascaplysin on the expression of P-selectin and the activation of GPIIb/IIIa on platelets. For this purpose, washed platelets were incubated with concentrations of 2–10 μM fascaplysin, stimulated with the three agonists PAR-1-AP, adenosine diphosphate (ADP) and phorbol-12-myristate-13-acetate (PMA) and analyzed by flow cytometry. We found that fascaplysin does not affect the expression of P-selectin after PAR-1-AP and ADP stimulation (Figure 2A,C). Moreover, only the higher dose of 10 μM fascaplysin significantly inhibited P-selectin expression on PMA-activated platelets (Figure 2E). In contrast, fascaplysin markedly suppressed the activation of GPIIb/IIIa, as indicated by reduced PAC-1 binding levels after PAR-1-AP, ADP or PMA stimulation (Figure 2B,D,F).

### 2.3. Effect of Fascaplysin on Platelet Aggregation

**Figure 3:**
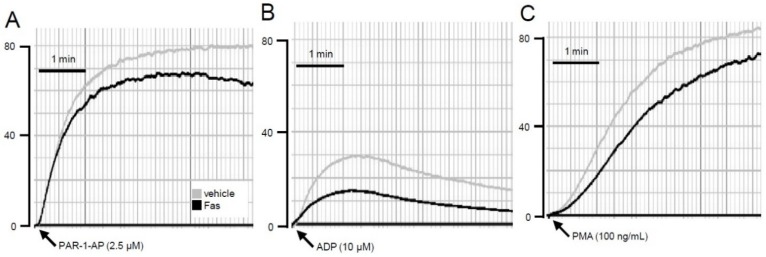
Effect of fascaplysin on platelet aggregation. (**A**–**C**) Typical diagrams of aggregation results for WP, which were incubated with 10 μM fascaplysin (black lines) or vehicle (grey lines) for 0.5 h prior to the stimulation with PAR-1-AP (**A**); ADP (**B**); or PMA (**C**).

Based on our finding that fascaplysin suppresses the activation of GPIIb/IIIa, we next analyzed the aggregation capacity of fascaplysin-treated washed platelets. As expected, treatment of washed platelets with 10 μM fascaplysin resulted in an attenuated agonist-induced platelet aggregation (Figure 3A–C).

### 2.4. Effect of Fascaplysin on PLA Formation

**Figure 4:**
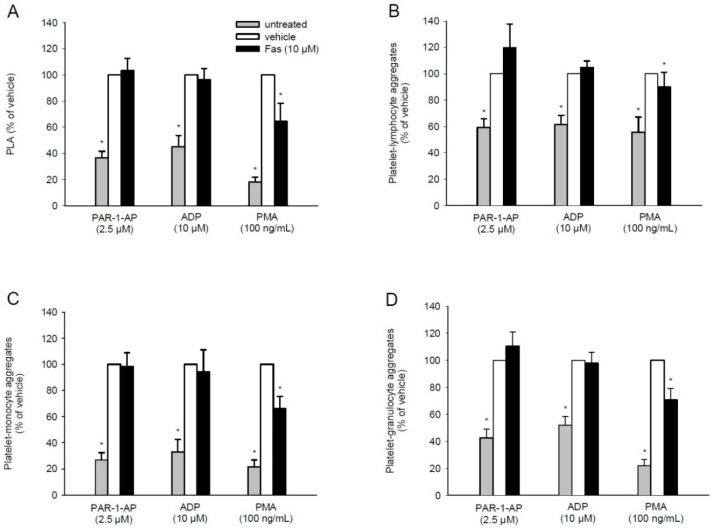
Effect of fascaplysin on platelet-leukocyte aggregates (PLA) formation. (**A**–**D**) Whole blood was incubated with 10 μM fascaplysin (black bars, *n* = 6) or vehicle (DMSO, white bars, *n* = 6) for 0.5 h followed by stimulation with PAR-1-AP, ADP or PMA. Data are given in % of vehicle. Unstimulated whole blood served as negative control (grey bars, *n* = 6). Platelets bound to all leukocytes (**A**) or lymphocytes (**B**); monocytes (**C**) and granulocytes (**D**) were assessed by flow cytometry using double fluorescence staining (CD45/CD42b). Mean ± SEM. * *p* < 0.05 *vs.* vehicle.

Besides the main initiator P-selectin, PLA formation also depends on GPIIb/IIIa [26]. Based on our finding that fascaplysin markedly diminishes agonist-induced GPIIb/IIIa activation, we next analyzed by flow cytometry whether it also affects the interaction of platelets with leukocytes. We detected an increased number of platelets bound to leukocytes after PAR-1-AP and ADP stimulation, which, however, was not influenced by fascaplysin pre-treatment. In contrast, fascaplysin caused a significant reduction of PMA-induced PLA formation (Figure 4A).

To gain further insights into this process, the binding of platelets to individual leukocyte subpopulations was analyzed (Figure 4B–D). In line with other studies [21,27], agonist stimulation was predominantly observed in platelet-monocyte aggregates and platelet-granulocyte aggregates (Figure 4C,D). As expected, fascaplysin did not affect the binding of platelets to lymphocytes, monocytes or granulocytes following PAR-1-AP or ADP stimulation. However, the PMA-induced interaction of platelets with monocytes or granulocytes was significantly reduced in the presence of fascaplysin (Figure 4B–D).

### 2.5. Effect of Fascaplysin on CD11b Expression on Leukocytes

**Figure 5:**
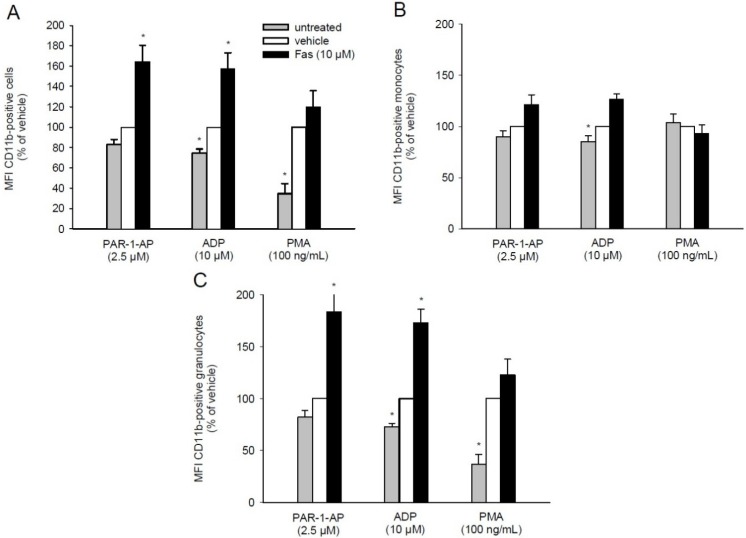
Effect of fascaplysin on leukocytic CD11b expression. (**A**–**C**) Whole blood was incubated with 10 μM fascaplysin (black bars, *n* = 6) or vehicle (DMSO, white bars, *n* = 6) for 0.5 h followed by stimulation with PAR-1-AP, ADP or PMA. Data are given in % of vehicle. Unstimulated whole blood served as negative control (grey bars, *n* = 6). Expression of CD11b on all leukocytes (**A**), monocytes (**B**) and granulocytes (**C**) were assessed by flow cytometry using double fluorescence staining (CD45/CD11b). Mean ± SEM. * *p* < 0.05 *vs.* vehicle.

The formation of PLA is not only dependent on the activation of platelets, but also on the expression of leukocytic adhesion molecules. In this context, several studies reported that the leukocytic integrin Mac-1 (CD11b/CD18) mediates the binding to GPIIb/IIIa on platelets via fibrinogen bridging [28,29,30]. Accordingly, we further assessed the expression of CD11b on leukocytes by means of flow cytometry. We found that fascaplysin treatment markedly increases the levels of CD11b following PAR-1-AP or ADP stimulation. However, PMA-induced CD11b levels were not affected by exposure of the cells to fascaplysin (Figure 5A). The additional analysis of different leukocyte subpopulations revealed that only granulocytes showed a similar expression pattern, but not monocytes (Figure 5B,C). This indicates that fascaplysin-induced up-regulation of CD11b expression primarily occurs in granulocytes.

### 2.6. Effect of Fascaplysin on Thrombus Formation and Tail-Vein Bleeding Time

**Figure 6:**
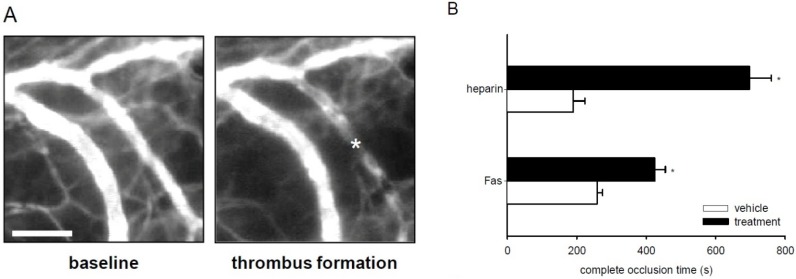

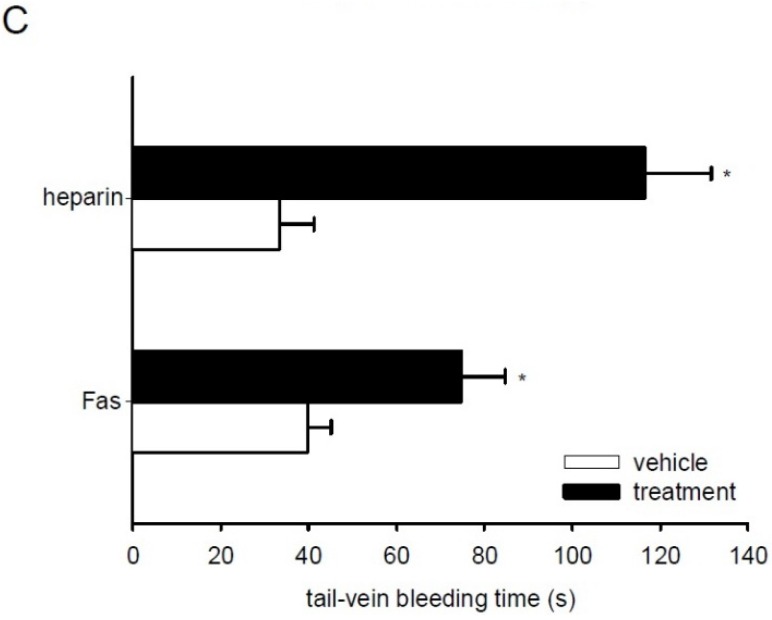
Effect of fascaplysin and heparin on thrombus formation and tail-vein bleeding time. (**A**) Intravital fluorescent microscopic images of a postcapillary venule in the dorsal skinfold chamber of a vehicle-treated BALB/c mouse before (baseline) and after photochemically induced thrombus formation (asterisk). Blue-light epi-illumination with contrast enhancement by 5% fluorescein isothiocyanate (FITC)-labeled dextran 150,000. Scale bar: 50 μm. (**B**) Complete occlusion time of postcapillary and collecting venules upon photochemically induced thrombus formation in dorsal skinfold chambers of heparin-treated (upper black bar, *n* = 3), fascaplysin-treated (lower black bar, *n* = 6), and vehicle-treated BALB/c mice (upper white bar, saline, *n* = 3 and lower white bar, DMSO, *n* = 6), as assessed by intravital fluorescence microscopy. Mean ± SEM. * *p* < 0.05 *vs.* vehicle. (**C**) Tail-vein bleeding time of heparin-treated (upper black bar, *n* = 3), fascaplysin-treated (lower black bar, *n* = 6), and vehicle-treated BALB/c mice (upper white bar, saline, *n* = 3 and lower white bar, DMSO, *n* = 6). Mean ± SEM. * *p* < 0.05 *vs.* vehicle.

Finally, we tested the anti-thrombotic activity of fascaplysin and heparin (positive control) in the dorsal skinfold chamber model of photochemically induced thrombus formation [31]. For this purpose, we analyzed postcapillary and collecting venules with a diameter of 15–25 μm, which exhibited comparable centerline red blood cell (RBC) velocities and wall shear rates (Table 1). As expected, heparin effectively increased complete vessel occlusion time and tail-vein bleeding time (Figure 6B,C), indicating that the present experimental setting is suitable to measure anti-thrombotic effects of test compounds. In line with our *in vitro* results, we detected a significantly prolonged complete vessel occlusion time within dorsal skinfold chambers of fascaplysin-treated mice when compared to vehicle-treated controls (Figure 6A,B). Moreover, treatment with fascaplysin also increased the tail-vein bleeding time (Figure 6C).

**Table 1:** Diameter (μm), centerline red blood cell RBC velocity (μm/s) and wall shear rate (s^−1^) of postcapillary and collecting venules in dorsal skinfold chambers of fascaplysin (Fas)-treated (*n* = 6), heparin-treated (*n* = 3) and vehicle-treated (DMSO (*n* = 6) or saline (*n* = 3)) BALB/c mice directly before photochemically induced thrombus formation, as assessed by intravital fluorescence microscopy and computer-assisted image analysis.

	Vehicle (DMSO)	Fas	Vehicle (Saline)	Heparin
**diameter (μm)**	16.7 ± 0.6	18.9 ± 0.7	20.9 ± 1.3	18.9 ± 1.6
**centerline RBC velocity (μm/s)**	205.1 ± 62.2	230.9 ± 50.3	267.9 ± 45.9	219.3 ± 70.3
**wall shear rate (s^−1^)**	98.3 ± 25.9	97.7 ± 24.5	102.54 ± 32.8	92.8 ± 21.7

Mean ± SEM.

## 3. Discussion

The marine-derived kinase inhibitor fascaplysin targets fundamental cellular processes, such as proliferation, apoptosis and angiogenesis [1,2,3]. Accordingly, fascaplysin has been suggested as a candidate for the establishment of novel anti-tumor therapies [1,5]. For this purpose, it is, however, necessary to characterize in detail the activity spectrum of the compound. In this context, we herein demonstrate for the first time that fascaplysin affects the activation of platelets and their interaction with leukocytes, resulting in the suppression of hemostasis and thrombus formation.

Recently, it has been demonstrated that exposure of human leukemia HL-60 cells to fascaplysin leads to an inhibition of all major proteins of the PI3K/AKT/mTOR pathway [5]. Moreover, it is well known that PI3K is a crucial regulator of thrombus formation [7,8,32]. In line with these studies, our results now show that fascaplysin reduces PI3K signaling in platelets and attenuates agonist-induced platelet aggregation. This may be explained by the fact that an attenuated PI3K activity deteriorates the affinity and avidity of GPIIb/IIIa for ligands, which is also called “inside-out signaling” [9,14]. However, we did not detect an effect of fascaplysin on the expression of P-selectin on PAR-1-AP- and ADP-stimulated platelets. This parallels findings of Kovacsovics *et al.* [33], demonstrating that treatment of platelets with the specific PI3K inhibitor wortmannin results in decreased levels of activated GPIIb/IIIa without affecting P-selectin expression.

Of interest, we additionally found that a higher dose of 10 μM fascaplysin significantly inhibited P-selectin expression on platelets stimulated by the strong PKC activator PMA. This indicates that fascaplysin also targets other kinases than PI3K. In this context, Anastassiadis *et al.* [34] tested fascaplysin against a panel of 300 kinases and detected an inhibitory effect of the compound on protein kinase C (PKC). PKC, which is downstream of phospholipase C (PLC), also mediates platelet aggregation by both GPIIb/IIIa activation and up-regulation of P-selectin expression. Hence, it may be assumed that besides the inhibition of PI3K a reduction of PKC activity contributed to the fascaplysin effects observed in the present study.

The formation of PLA is a characteristic event during thrombus formation, which is mainly mediated by the binding of P-selectin on platelets to leukocytic PSGL-1 [16,22,35]. Besides, it has been reported that GPIIb/IIIa binds to the leukocytic integrin Mac-1 (CD11b/CD18) via fibrinogen as a bridging molecule [26,36,37]. The herein observed reduction of activated GPIIb/IIIa on fascaplysin-treated platelets suggests that the latter binding mechanism may have affected the formation of PLA. However, this was not the case when platelets were stimulated with PAR-1-AP or ADP. This interesting finding prompted us to further analyze the expression of CD11b on leukocytes. A small fraction of the total CD11b content is stored intracellularly and is rapidly up-regulated as a result of different types of stimuli [38]. Previous studies indicate that a reduced GPIIb/IIIa activation may represent such a stimulus [28,39]. In fact, Hu *et al.* [28] demonstrated that this leads to an enhanced level of CD11b on leukocytes, which, in turn, may compensate the down-regulation of activated GPIIb/IIIa. In line with this point of view, we measured increased levels of leukocytic CD11b in fascaplysin-treated whole blood after PAR-1-AP or ADP stimulation.

In contrast, we detected a significantly lower rate of PLA following stimulation with the PKC activator PMA. This result may be caused by the aforementioned inhibitory effect of fascaplysin on PKC. In fact, PKC has been shown to mediate CD11b expression [38,40]. Accordingly, we did not detect a marked up-regulation of leukocytic CD11b expression in PMA-exposed whole blood when compared to PAR-1-AP- and ADP-treated blood. Thus, it is tempting to speculate that these lower levels of CD11b may have not been sufficient to compensate the fascaplysin-induced down-regulation of GPIIb/IIIa on platelets. The reduced formation of PLA may additionally be explained by the observation of Davenbeck *et al.* [16] that PSGL-1 is significantly decreased on human leukocytes following PMA stimulation.

Finally, we confirmed the observed *in vitro* effects of fascaplysin on platelet aggregation and PLA formation in an *in vivo* model of photochemically induced thrombus formation [31,41]. Thrombus formation was induced in postcapillary and collecting venules in mouse dorsal skinfold chambers by administration of fluorescein isothiocyanate (FITC)-labeled dextran and transmural blue light exposure, which results in endothelial injury by local generation of reactive oxygen species [41]. Noteworthy, the microvessels of fascaplysin-treated and vehicle-treated animals did not exhibit any differences in microhemodynamic parameters, indicating that thrombus formation could be analyzed in both groups under comparable conditions. In accordance to our *in vitro* results we found that complete vessel occlusion time was significantly prolonged by treatment with fascaplysin. In contrast to other anti-platelet agents, such as aegyptin, dipetalodipin and triplatin [42,43], fascaplysin further affected primary hemostasis during tail-vein bleeding. Moreover, fascaplysin showed a lower anti-thrombotic efficiency in our *in vivo* experiments when compared to heparin. Therefore, this compound may not be suitable as an anti-thrombotic drug for future clinical use. However, its anti-thrombotic activity should be considered as a potential side effect when using fascaplysin as an anti-cancer agent.

In summary, the present study demonstrates that fascaplysin reduces GPIIb/IIIa activation and aggregation of platelets. This prevents thrombus formation and increases bleeding time. These results indicate for the first time that fascaplysin exerts anti-thrombotic activity, which represents a novel mode of action in the pleiotropic activity spectrum of this marine-derived kinase inhibitor.

## 4. Experimental Section

### 4.1. Chemical and Biological Reagents

Fascaplysin was purchased from Santa Cruz Inc. (Heidelberg, Germany), calcein-AM from Molecular Probes (Eugene, OR, USA), heparin from Braun (Melsungen, Germany), ADP sodium salt, PAR-1-AP, PMA, dimethyl sulfoxide (DMSO), and FITC-labeled dextran 150,000 from Sigma-Aldrich (München, Germany), ketamine (Ursotamin^®^) from Pharmacia GmbH (Erlangen, Germany), xylazine (Rompun^®^) from Bayer (Leverkusen, Germany), and Endothelial Cell Basal Medium (C-22210) from PromoCell (Heidelberg, Germany).

### 4.2. Antibodies

The antibodies anti-CD11b (555388), anti-CD45 (3325829), anti-CD42b (555473), anti-CD62P (555524), anti-GPIIb/IIIa (PAC-1) (340507), anti-PI3K (p85) (610045) and IgG1-κ isotype control (555749) were purchased from BD Biosciences (Heidelberg, Germany). The antibodies phospho (*p*)-ERK (50011) and ERK (115799) were purchased from abcam (Cambridge, UK). The antibody α-tubulin (T6199) was purchased from Sigma-Aldrich (München, Germany). The antibody CDK4 (C-22) was purchased from Santa Cruz Inc. (Heidelberg, Germany).

### 4.3. Endothelial Cell Culture

HDMEC were purchased from PromoCell and cultivated in Endothelial Cell Basal Medium at 37 °C under a humidified 95% to 5% (*v*/*v*) mixture of air and CO_2_. Cells were passaged at a split ratio of 1:3 after reaching confluence.

### 4.4. Ethics Statement

For the isolation of platelet rich plasma (PRP), platelets and leukocytes, venous blood was drawn from six healthy volunteers after obtaining their written informed consent, and with the approval of the local ethics review board.

### 4.5. Preparation of Washed Platelets

Washed platelets were prepared as previously described [44]. Briefly, venous blood from six healthy voluntary donors was drawn into plastic syringes containing 0.1 volume of 3.2% trisodium citrate and centrifuged at 100× *g* for 20 min at room temperature. The supernatant (PRP) was collected and centrifuged at 2200× *g* for 3 min. Subsequently, the supernatant was discarded and the pellet was washed twice in a cation-free HEPES-Tyrode buffer (pH 6.5) containing 1 mM EGTA. Afterwards, the pellet was resuspended in HEPES-Tyrode buffer (pH 7.4) containing 1.25 mM CaCl_2_ and 6.25 mg/mL BSA. Platelets were counted and adjusted to the concentration of 3 × 10^8^ platelets/mL.

### 4.6. Western Blot Analysis

Protein levels of CDK4 and p85 were analyzed in untreated HDMEC and washed platelets. In additional experiments, washed platelets were treated for 30 min with different concentrations of fascaplysin or vehicle (DMSO) and then stimulated with PAR-1-AP (2.5 μM) for 5 min. The cells were centrifuged for 3 min by 4 °C and lysed on ice for 10 min in lysis buffer (RIPA buffer: 50 mM Tris-HCl, pH 7.2, 0.15 M NaCl, 1.0 mM EDTA, 0.1% SDS, 1.0% Triton X-100, 1.0% sodium deoxycholate, and phosphatase inhibitor). Cell extracts were separated through a 10% SDS polyacrylamide gel and immunoblotted with specific antibodies. Protein expression was visualized by luminol-enhanced chemiluminescence (ECL; GE-Healthcare, Freiburg, Germany) and exposure of the membranes to a blue light sensitive autoradiography film (Hyperfilm ECL, GE-Healthcare, Freiburg, Germany).

### 4.7. Platelet Aggregation

Platelet aggregation was measured using a Born aggregometer (APACT 4SPlus, Rolf Greiner Biochemica, Flacht, Germany) at 37 °C under stirring conditions (700–1000 U/min). Aliquots of 180 μL washed platelets or HEPES-Tyrode buffer (pH 7.4) were incubated with the indicated concentrations of fascaplysin or vehicle (DMSO) for 30 min at 37 °C. Fascaplysin and DMSO were prediluted in HEPES-Tyrode buffer (pH 7.4) to lower the DMSO concentration. After incubation, HEPES-Tyrode buffer (pH 7.4) was used to calibrate the aggregometer to 0% before measuring and unstimulated washed platelets were used to calibrate up to 100% aggregation. Vehicle measuring was performed in all repeats. Washed platelets were stimulated with the agonists 2.5 μM PAR-1-AP, 10 μM ADP or 100 ng/mL PMA and change in light transmission was measured for 5 min.

### 4.8. PLA

PLA were induced as previously described [21]. Briefly, venous blood from six healthy human donors was drawn into plastic syringes containing 0.1 volume of 3.2% trisodium citrate. Subsequently, 10 μL blood was added to 40 μL of HEPES-Tyrode buffer (pH 7.4), containing appropriate concentrations of phycoerythrin (PE)-labeled anti-PE-CD42b and FITC-labeled anti-CD45 antibodies. The samples were treated with the indicated concentrations of fascaplysin or vehicle (DMSO) and incubated under static conditions for 15 min. After stimulation with ADP (10 μM), PMA (100 ng/mL) or PAR-1-AP (2.5 μM) for 20 min, the samples were fixed by adding 1% formalin (300 μL) and analyzed by flow cytometry.

### 4.9. Flow Cytometry

The viability of washed platelets was analyzed by flow cytometry, as previously described [45]. For this purpose, washed platelets were treated for 30 min with different concentrations of fascaplysin or vehicle (DMSO). Subsequently, 1 μL of calcein-AM (8 μM) and saturating concentrations of PE-labeled anti-CD42b antibody were added to 40 μL of washed platelets and incubated for 0.5 h. The appearance of double-colored events was then determined in a FACScan flow cytometer (Becton Dickinson, San Jose, CA, USA) using CellQuest software.

Moreover, washed platelets were treated for 30 min with different concentrations of fascaplysin or vehicle (DMSO) followed by ADP-stimulation (10 μM), PMA (100 ng/mL) or PAR-1-AP-stimulation (2.5 μM) for 5 min. Aliquots (10 μL) of washed platelets were incubated with saturating concentrations of FITC-labeled anti-PAC-1 antibody, PE-labeled anti-CD62P (P-selectin) antibody or control antibody (isotype control) for 0.5 h. Afterwards the aliquots were fixed in 1% formalin for 10 min at 4 °C and were analyzed by flow cytometry. The fluorescence of 50,000 platelets was analyzed in the FACScan flow cytometer (Becton Dickinson, San Jose, CA, USA) using CellQuest software. The expression of activated GPIIb/IIIa and P-selectin was determined by analyzing the mean fluorescence intensity in the FL2 channel.

In a subset of experiments, double fluorescence staining was performed in whole blood to analyze PLA as previously described [21]. Briefly, samples were analyzed in the FACScan flow cytometer using the CellQuest software. For the analysis, a quadrant was set in the dot plot of respective channels on non-stimulated platelets. The appearance of double-colored events in the upper right quadrant was determined. Then, CD45-labeled lymphocytes, monocytes and granulocytes were separately gated according to their size and granularity and the mean fluorescence intensity of PE-labeled anti-CD42b was analyzed.

In addition, CD11b expression was determined as mean fluorescence intensity (MFI) in CD45-labeled total leukocytes, monocytes or granulocytes.

### 4.10. Animals

BALB/c mice with a body weight of 20–25 g were used for the experiments. They were housed in a temperature-controlled environment under a 12 h/12 h light-dark cycle and received standard pellet food (Altromin, Lage, Germany) and water *ad libitum*. All experiments were approved by the local governmental animal care committee (*Landesamt für Verbraucherschutz*, *Abteilung C Lebensmittel- und Veterinärwesen*, Saarbrücken, Germany; Permit Number: 15/2014) and were conducted in accordance with the European legislation on protection of animals (Guide line 2010/63/EU) and the NIH Guidelines for the Care and Use of Laboratory Animals.

### 4.11. Photochemically Induced Thrombus Formation

Mouse dorsal skinfold chambers were used to investigate *in vivo* the effect of fascaplysin on photochemically induced thrombus formation [31]. For this purpose, 18 BALB/c mice were anesthetized with an intraperitoneal injection (i.p.) of 75 mg/kg ketamine and 15 mg/kg xylazine and dorsal skinfold chambers were implanted, as previously described [46]. To avoid alterations of the microcirculation due to anesthesia or surgical trauma, the mice were allowed to recover from the implantation procedure for 72 h. Subsequently, animals were treated with 5 mg/kg fascaplysin i.p. (*n* = 6) or vehicle i.p. (DMSO; *n* = 6) 19 h and 1 h before photochemically induced thrombus formation. The dose of 5 mg/kg fascaplysin has previously been shown in tumor studies to effectively inhibit tumor growth *in vivo* without inducing toxic side effects [47,48]. In control experiments, animals were treated with an intravenous injection (*i.v.*) of 100 IU/kg heparin (*n* = 3) or vehicle (saline; *n* = 3) 15 min before photochemically induced thrombus formation.

For *in vivo* microscopic observation, mice were immobilized on a Plexiglas stage and the dorsal skinfold chamber was attached to the microscopic stage. After retrobulbary i.v. injection of 0.05 mL 5% FITC-labeled dextran 150,000 for contrast enhancement by staining of blood plasma, intravital epi-illumination fluorescence microscopy was performed using a Zeiss microscope (Zeiss, Oberkochen, Germany) with a 100 W mercury lamp attached to a blue filter. The microscopic images were recorded by a charge-coupled device video camera (FK6990; Pieper, Schwerte, Germany) and transferred to a monitor (Trinitron; Sony, Tokyo, Japan) and DVD system (DVD-HR775; Samsung, Eschborn, Germany) for off-line evaluation [31].

Using a ×20 long distance objective (Achroplan 0.50 W; Zeiss, Oberkochen, Germany), baseline blood flow was monitored in individual venules (diameter range: 15–25 μm; *n* = 4 per chamber). Subsequently, thrombus formation was photochemically induced by continuous local exposure of the vessels to filtered light (450–490/>520 nm excitation/emission wavelength) with a ×63 water immersion objective (Achroplan 0.95 W; Zeiss, Oberkochen, Germany) [41,49].

Quantitative off-line analyses of the microscopic images were performed using the software package CapImage (Zeintl, Heidelberg, Germany). Diameters, centerline RBC velocity and wall shear rate were determined in venules prior to thrombus induction. Diameters (*d*) were measured in μm perpendicularly to the vessel path. Centerline RBC velocity (*v*, given in μm/s) was analyzed using the line shift method [50] and wall shear rate (*y*, given in s^−1^) was calculated based on the Newtonian definition: *y* = 8 × *v*/*d*. The kinetics of thrombus formation was assessed by measuring the time (given in s) until sustained cessation of blood flow due to complete vessel occlusion.

### 4.12. Tail-Vein Bleeding Time

At the end of the dorsal skinfold chamber experiments, tail-vein bleeding time was determined as a parameter of primary hemostasis, as previously described [51]. Briefly, an incision was made over a lateral tail vein at a constant position of the tail. Subsequently, the tail was immersed in saline (37 °C). The time from incision to complete cessation of the blood stream was measured as bleeding time. At the end of the *in vivo* experiments, the animals were sacrificed with an overdose of the anesthetics.

### 4.13. Statistical Analysis

After testing the data for normal distribution and equal variance, differences between the groups were analyzed by the unpaired Student’s *t*-test (SigmaStat; Jandel Corporation, San Rafael, CA, USA). All values are expressed as means ± SEM. Statistical significance was accepted for a value of *p* < 0.05.
